# Clinical trials from the patient perspective: survey in an online patient community

**DOI:** 10.1186/s12913-017-2090-x

**Published:** 2017-02-27

**Authors:** Pronabesh DasMahapatra, Priya Raja, Jeremy Gilbert, Paul Wicks

**Affiliations:** PatientsLikeMe Inc, 155 Second Street, Cambridge, MA 02141 USA

**Keywords:** Clinical trials, Engagement, Recruitment, Retention, Patient-centeredness, Trial design

## Abstract

**Background:**

Developing new medicines relies on the successful conduct of clinical trials. As trial protocols become more arduous, it becomes harder to recruit and retain patient volunteers, although recent efforts such as OMERACT and I-SPY2 show that partnering with patients can be beneficial. We sought to describe drivers and barriers to trial participation, as well as condition-specific trial preferences.

**Methods:**

An online survey was fielded via the patient-powered research network PatientsLikeMe to 1,621 members living with nine selected chronic health conditions. Questions included demographics, trial experience, reasons for non-participation, questions relating to aspects of trial design, and an adaptation of the Net Promoter Score (NPS) for trial satisfaction.

**Results:**

Mean age of respondents was 55 years; most patients were white (93%), female (67%), and living in the United States (72%). Primary conditions were MS (21%), Parkinson’s (20%), fibromyalgia (15%), ALS (10%), type 2 diabetes (10%), rheumatoid arthritis (RA, 8%), epilepsy (8%), major depressive disorder (MDD, 5%) and systemic lupus erythematosus (SLE, 3%). Most patients had not discussed a trial with their physician and only 21% had ever enrolled, with rates highest in ALS (36%), Parkinson’s disease (36%) and MS (20%) and lowest among SLE (9%), MDD (11%) and Fibromyalgia (11%). Common reasons for non-participation were eligibility criteria, inconvenience of travel and concerns about side effects. NPS suggested that many patients were unsatisfied; patients with lupus, epilepsy, RA, and fibromyalgia reported negative scores, i.e. they would dissuade other patients like them from taking part in trials. The most important considerations in trial participation were the opportunity to improve one’s own health and that of others, the reputation of the institution, and having medical bills covered in case of injury. Least important were remuneration and possibility of receiving a placebo. ALS patients were more willing to tolerate undesirable aspects of trials.

**Conclusions:**

Most patients are willing to enroll yet very few are invited. When they do, trial participation is often burdensome, but patients are willing to help improve their design. Researchers should let patients help design better trials to overcome recruitment and retention issues and hasten the development of new medicines.

**Electronic supplementary material:**

The online version of this article (doi:10.1186/s12913-017-2090-x) contains supplementary material, which is available to authorized users.

## Background

The development of new and innovative medicines is being hampered by the rising cost of clinical trials [1], with one estimate for the cost of bringing a drug to market as $2.6 billion, a 145% increase from 2003 [2]. Despite energy and resources devoted to study planning, protocol development, and physician reimbursement, many studies fail to recruit enough patients to be adequately powered, and a subset even fail to recruit *any* patients [3–5]. Even once recruited, patients may still choose to prematurely drop out of a trial, compromising the study’s validity [6]. Tools and strategies that overcome challenges to recruitment and retention have the potential to reduce costs and so accelerate research, but this first requires a thorough understanding of the potential barriers. These include (1) organizational factors, (2) healthcare professional factors, and (3) patient factors [7, 8].

Many patients harbor misconceptions about trials; in a survey of nearly 6,000 patients, 37% thought their medical care would be better if they *did not* enroll in a trial, and 22% believed enrolling in a clinical trial would lead to them being “treated like a guinea pig” [9]. Such fears are not completely unfounded given that that participating in a trial is increasingly burdensome, with the median number of study procedures that patients must endure rising from 20 per protocol in 2000–2003 to 30 in 2008–2011 [10]. Listening effectively to patients at early stages may uncover patient-facing obstacles, facilitating effective planning and minimizing burden. Patient-centered approaches to study design and execution might even yield more successful studies [11] and methods to support higher levels of patient engagement have been successfully introduced by research groups such as the OMERACT group [12] and the I-SPY2 trial [13]. In both examples, patients helped resolve research challenges by (1) addressing undesirable aspects of trials, (2) surfacing new perspectives to researchers, (3) interacting directly with researchers, and (4) co-producing new resources that patients may find beneficial before, during and after the trials [14].

Such examples are few and far between, however, and the potential to prevent costly and avoidable protocol amendments [15] has stimulated interest to systematically involve patients in trial designs on a wider scale. However, deployment of patient-centered engagement models are challenging to implement and sustain over a period of time. Patient-powered research networks (PPRNs) might offer one useful model to streamline real-time patient input into clinical trial planning, having the advantage of inherent scalability, reaching patients in their homes, and having lower barriers than traditional methods to ask multiple rounds of iterative questions [16]. We sought to conduct a pilot study with one PPRN, PatientsLikeMe (PLM), to understand motivations and barriers to trial participation, and to identify opportunities to enhance the clinical trial experiences of patients with chronic disease. As a pilot study, exploratory objectives were to (1) identify socio-demographic factors associated with trial participation; and (2) explore the differences in perceptions and attitudes about trials by type of chronic conditions. The long-term objective of this study was to build a systematic infrastructure to engage patients in trial design so that academics and pharmaceutical companies investigating new drugs could quickly draw upon evidence to optimize the design of each unique trial.

## Methods

### Setting and study design

Members of PatientsLikeMe share data about their conditions, treatments, symptoms and comorbidities through structured data collection in order to connect with other patients like them, manage their condition, and contribute to scientific research [17]. An online survey was administered to members of the PLM website between February and March 2014. The survey items were developed with the intention of elucidating patient perceptions and experience of clinical trials. Development and usability pre-testing were performed iteratively by the authors. The survey was then reviewed for editorial and technical suggestions by the Quality Assurance staff at PLM. Invitations were sent via private electronic message to an unrestricted convenience sample of members. Completion of the entire survey took about 15 min and was shorter for those who skipped or branched out of sub-sections. Given that participation in the survey was voluntary and members were not remunerated for their participation, patients were unlikely to provide spurious data, although no formal validation of data accuracy was undertaken. Survey responses were collected using a proprietary survey tool and stored in a secure database.

Independent ethics review was not sought as members (patients) of the PLM community are informed of their involvement in research activities via the user agreement and privacy policy before joining the site. Further, the study falls under Office for Human Research Protections (OHRP) Exempt Categories 45 CFR 46.101(B) i.e., research involving the use of educational tests (cognitive, diagnostic, aptitude, achievement), survey procedures, interview procedures or observation of public behavior.

### Measures

Demographic variables included age, sex, ethnicity, race, country of residence, and primary medical condition. The survey included three major sections: (1) past experience with clinical trials; (2) attitudes and interest towards clinical trials; and (3) perceived factors that might influence clinical trial participation. Skip-logic and conditional branching were embedded within the survey. Response options varied by the type of questions and ranged from numerical, categorical, ordinal (Likert scale) to open ended comments. Multiple-choice questions with “select all that apply” response choice were randomized to avoid primacy and recency effects. An adaptation of Net Promoter Score (NPS), a single item measure of consumer satisfaction [18] was used to assess trial performance across conditions. NPS has been used as an overarching measure of patient experience with healthcare delivery. Patients were asked “How likely would you be to recommend taking part in this specific trial to another eligible patient like you?” (0–6: detractors, 7–8: passive and 9–10: promoters). A NPS score was calculated as the percentage of respondents who were promoters and subtracting the percentage that were detractors. A copy of the survey as fielded to participants is supplied in Additional file 1: Appendix E-1.

### Participants

Participants were selected from the PLM database of patients with the nine most frequently reported “primary” conditions (i.e. a member’s chief complaint). The inclusion criteria were: (1) Registered members of the PLM website, (2) aged 18 years or over, (3) website activity within the past 90 days, (4) Reporting one of the following 9 primary medical conditions - amyotrophic lateral sclerosis (ALS), type 2 diabetes mellitus (T2DM), epilepsy, fibromyalgia, multiple sclerosis (MS), major depressive disorder (MDD), Parkinson’s disease (PD), rheumatoid arthritis (RA), or systemic lupus erythematosus (SLE) on their PLM profile.

### Data analyses

Analyses were only conducted in patients who completed the entire survey. Duplicate entries were removed from analyses. Descriptive statistics were computed for demographic variables and presented as mean (SD) for continuous variable age and frequency (%) for categorical variables sex, race, region, ethnicity and primary medical condition. Difference in demographic distribution between groups (e.g., completers vs. non-completers, trial experience vs. no prior trial experience) were tested using *t*-test for age and *χ* ^2^ test for categorical variables. Questions pertaining to awareness, participation and interest in clinical trials were reported as frequency (%) for categorical variables. The measure of association between socio-demographic factors and trial participation was presented as Odds ratio (OR). Data analyses were performed for all survey completers as well as stratified by the primary condition. Differences between primary conditions were tested by using the MULTTEST procedure with bootstrap adjusted *p*-values for pairwise comparisons, where appropriate. Data analyses were performed in SAS version 9.4 (SAS Institute Inc., Cary, NC). The level of significance was set at α = .05. A-priori power estimation was not performed.

## Results

### Participant characteristics

**Fig. 1:**
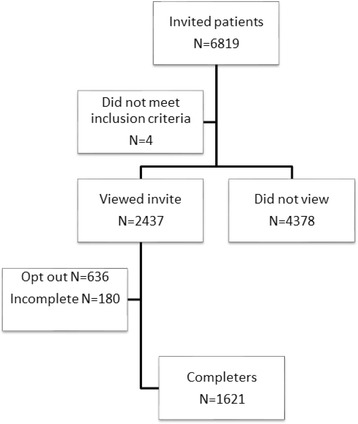
Flow of participation in the survey. Six-thousand eight-hundred and nineteen patients (*n* = 6,819) were invited of which *n* = 2,437 viewed the invite and *n* = 1,621 completed the survey, yielding view rate (n of views/n of eligible invites), participation rate (n of participation/n of views) and completion rate (n of completers/n of participation) of 36%, 74 and 90%, respectively

Six thousand eight hundred and nineteen (*n* = 6,819) unique members were invited to participate in the survey of which 6,815 met the eligibility criteria. Two thousand four hundred and thirty-seven (*n* = 2,437) viewed the invitation resulting in a view rate (n of views/n of eligible invites) of 36%. Among those who viewed the invite (*n* = 2,437), 1,621 completed the survey, 636 opted out, and 180 provided partial data, yielding participation rate (n of participation/n of views) and completion rate (n of completers/n of participation) of 74% and 90%, respectively (Fig. 1). The completion rate was in line with Internet-based surveys of similar length (approximately 70%) [19].

Among completers, the mean age of members at the time of the survey was 55 years (SD: 11 years); 67% (*n* = 1,078) were female, 93% were white (*n* = 1,473). The primary conditions cited by completers were multiple sclerosis (21%, *n* = 343), Parkinson’s disease (20%, *n* = 319), fibromyalgia (15%, *n* = 241), amyotrophic lateral sclerosis (10%, *n* = 160), type 2 diabetes mellitus (10%, *n* = 158), rheumatoid arthritis (8%, *n* = 125), epilepsy (8%, *n* = 131), major depressive disorder (5%, *n* = 91) and systemic lupus erythematosus (3%, *n* = 53) (Table 1). The majority of members were from the United States (72%, *n* = 1,168), United Kingdom (10%, *n* = 158), Canada (8%, *n* = 134) and Australia (3%, *n* = 50).

**Table 1:** Characteristics of study completers vs non-completers

	Total (*N* = 6,815)	Completers (*N* = 1,621)	Non-completers (*N* = 5,194)	Test Statistic	*p*-value
N	6,815	1,621	5,194		
Age (years) ^a^				7.7	<0.0001
Mean ± SD	53 ± 13	55 ± 11	52 ± 13		
Unknown, N (%)	231 (3%)	0 (0%)	231 (4%)		
Sex, N (%)^a^				0.0	0.989
Female	4,390 (65%)	1,078 (67%)	3,312 (64%)		
Male	2,210 (32%)	543 (33%)	1,667 (32%)		
Unknown	215 (3%)	0 (0%)	215 (4%)		
Primary Condition, N (%)				283.1	<0.0001
ALS	833 (12%)	160 (10%)	673 (13%)		
T2DM	1,249 (18%)	158 (10%)	1091 (21%)		
Epilepsy	556 (8%)	131 (8%)	425 (8%)		
Fibromyalgia	665 (10%)	241 (15%)	424 (8%)		
MS	1,000 (15%)	343 (21%)	657 (13%)		
MDD	543 (8%)	91 (5%)	452 (9%)		
PD	998 (15%)	319 (20%)	679 (13%)		
RA	624 (9%)	125 (8%)	499 (10%)		
SLE	346 (5%)	53 (3%)	293 (5%)		

*SD* Standard deviation

^a^ Only available data (excluding unknown) used for statistical comparison between completers and non-completers

Using available profile data from non-completers, completers were around 3 years older than non-completers (*t*-test = 7.7, *p* < 0.0001). The groups also differed significantly on primary condition (*χ* ^2^ = 283.1, *p* < 0.0001). Of note, the percentage of completers relative to non-completers were higher for MS (21% vs. 13%), fibromyalgia (15% vs. 8%) and PD (20% vs 13%); and lower for T2DM (10% vs. 21%) and MDD (5% vs. 9%) (Table 1). The demographics of trial completers broken down by condition are shown in Table 2.

**Table 2:** Demographic Characteristics by Primary Condition among Completers

	N	Age (Mean ± SD)	Sex (Female)	Race (White)	Trial experience
ALS	160	58 ± 10	65 (41%)	152 (95%)	57 (36%)
T2DM	158	58 ± 9	79 (50%)	138 (87%)	20 (13%)
Epilepsy	131	47 ± 12	83 (63%)	115 (88%)	25 (19%)
Fibromyalgia	241	52 ± 10	220 (91%)	228 (95%)	26 (11%)
MS	343	51 ± 9	246 (72%)	300 (87%)	67 (20%)
MDD	91	44 ± 12	58 (64%)	83 (91%)	10 (11%)
PD	319	63 ± 8	171 (54%)	306 (96%)	116 (36%)
RA	125	54 ± 10	105 (84%)	107 (86%)	20 (16%)
SLE	53	49 ± 12	51 (96%)	44 (83%)	5 (9%)
All Completers	1,621	55 ± 11	1078 (67%)	1473 (91%)	346 (21%)

### Patient experience with trials

**Fig. 2:**
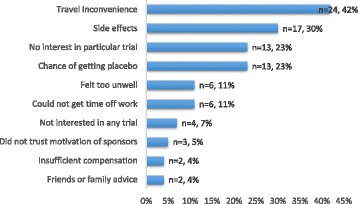
Reasons for declining trial participation amongst those that declined. The most common reasons were travel inconvenience, concerns over side effects and no interest in particular trial. Mistrust of sponsors, insufficient compensation and advice from family/friends were the least cited reasons. Note: Categories not mutually inclusive; percentages sum to greater than 100%

**Fig. 3:**
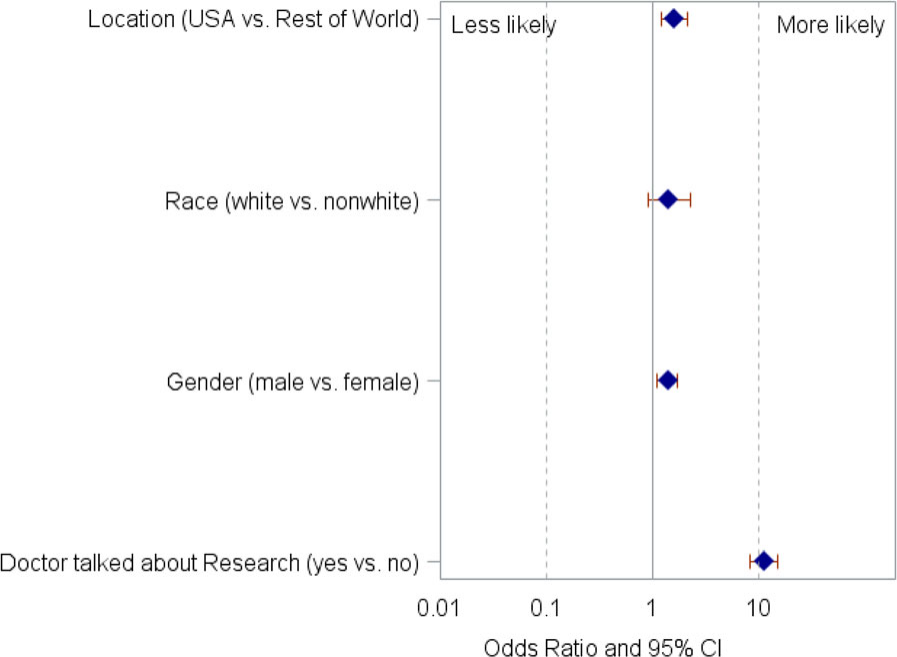
Factors associated with Participation in Trials. Socio-demographic factors that were associated with trial participation were: discussing medical research with physician (OR 11.2, 95% CL 8.3–15.1), US residence (OR 1.6, 95% CL 1.2–2.1), and male sex (OR 1.4, 95% CL 1.1–1.7)

In our study population, 39% (*n* = 630/1621) of patients reported having discussed medical research with their physician and 31% (*n* = 496/1621) reported being invited to participate in a clinical trial. One-fifth (21%) of patients had ever enrolled in a clinical trial (i.e. were “trial experienced”) (*n* = 346/1,621, Table 2) with 65% of the trial experienced subgroup (*n* = 222/346) completing one or more trial(s). About a quarter, 24% (*n* = 82/346) were enrolled in a trial at the time of the survey and 12% (*n* = 42/346) had withdrawn from a trial before its conclusion. Among trial experienced patients, the mean age at the time of the survey was higher (57 years vs. 54 years) compared to those without prior trial experience. The proportion of trial experienced patients was highest among patients with ALS (36%, *n* = 57/160), Parkinson’s disease (36%, *n* = 116/319) or MS (20%, *n* = 67/343); and lowest among those with SLE (9%, *n* = 5/53), MDD (11%, *n* = 10/91) or fibromyalgia (11%, *n* = 26/241) (Table 1). Among those invited to a trial some did not meet the eligibility criteria (19%, *n* = 93/496) and a few declined to participate (11%, *n* = 57/496). The reasons cited for declining participation in trials include, in descending order: inconvenience of travel (42%, *n* = 24/57), concerns about side effects (30%, *n* = 17/57), chance of getting a placebo (23%, *n* = 13/57) and having no interest in the particular trial (23%, *n* = 13/57) (Fig. 2). Socio-demographic factors that were associated with trial participation were: discussing medical research with physician (OR 11.2, 95% CL 8.3–15.1), US residence (OR 1.6, 95% CL 1.2–2.1), and male sex (OR 1.4, 95% CL 1.1–1.7) (Fig. 3).

**Fig. 4:**
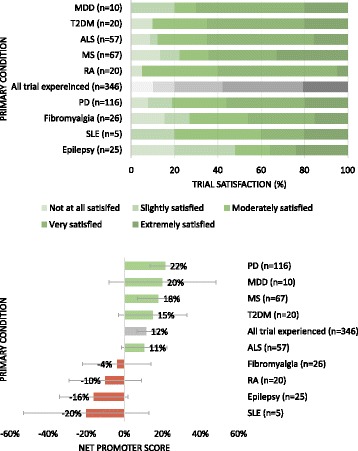
**a**, **b**. Trial satisfaction (**a**) and Net Promoter Score (**b**) by primary condition (patient’s chief complaints). Patients with Parkinson’s disease and MS were likely to endorse trials to other patients; in RA and Epilepsy participants were more likely to dissuade other patients like them from taking part in trials. Overall, 58% patients were “Very” to “Extremely” satisfied with the trials they partook in. Error bars represent 95% Confidence Intervals (95% CI)

The majority of trial experienced participants (58%, *n* = 200) reported being “extremely” to “very satisfied” with their participation while a fifth (20%) were only “slightly” or “not at all” satisfied. An overall trial satisfaction as measured by NPS, revealed that 46% (*n* = 159/346) were classified by the scoring system as “promoters” and 34% (n = 119/346) were “detractors” yielding an average score (% promoters - % detractors) of 12% (95% CI: 7% to 17%. Fig. 4). NPS was higher in patients with Parkinson’s disease (NPS: 22%, 95% CI: 14% to 30%) and MS (NPS: 18%, 95% CI: 7% to 29%); and lower in RA (NPS: −10%, 95% CI: −29% to 9%) and Epilepsy (NPS: −16%, 95% CI: −34% to 2%), where some participants would actively dissuade other patients like them from taking part in trials.

At the time of the survey, the mean age of participants who withdrew before trial conclusion was 58 years. Trial withdrawal was higher in females (16%, *n* = 33/211) relative to males (7%, *n* = 9/135). The proportion of trial withdrawal was highest among patients with SLE (40%, *n* = 2/5) and MS (21%, *n* = 14/57). NPS was also notably lower in patients who withdrew from trials (NPS: −28%, 95% CI: −52% to −24%) relative to those who completed or were in a trial at the time of the survey (NPS: 18%, 95% CI: 13% to 23%).

### Overall attitudes toward trials

Participants expressed a high degree of interest in clinical trials. Specifically, 88% (*n* = 1,434/1,621) agreed they were “Interested in learning more about taking part in trials” and 80% (*n* = 1,288/1,621) agreed to “I would like to take part in a trial in the next 12 months”. Finally, 93% (*n* = 1,503/1,621) agreed that they would be “Interested in helping researchers to design better trials”, suggesting an opportunity to engage with this population again on a trial-by-trial basis to develop better protocols and engagement methods.

When asked to rate factors they might consider before joining a clinical trial, participants cited the following factors as “very” to “somewhat” important (in descending order of ratings): (1) opportunity to improve health of others (98%, *n* = 1,593/1,621) and self (98%, *n* = 1,586/1,621), (2) reputation of the institution (97%, *n* = 1,578/1,621), and (3) covering medical bills in case of a trial related injury (96%, *n* = 1,551/1,621). The least important considerations were: (1) remuneration for participation (46%, *n* = 734/1,621), and (2) receiving a placebo (64%, *n* = 1,046/1,621). Perceived importance of these factors across conditions and significant pairwise differences are shown in Table 3.

**Table 3:** Perceived importance of factors for trial participation by condition

	ALS (N=160)	Diabetes Type II (N=158)	Epilepsy (N=131)	Fibromyalgia (N=241)	MS (N=343)	MDD (N=91)	PD (N=319)	RA (N=125)	SLE (N=53)	All Completers (N=1,621)
Opportunity to improve health of others	156 (98%)	156 (99%)	126 (96%)	237 (98%)	340 (99%)	90 (99%)	315 (99%)	123 (98%)	50 (94%)	*1,593 (98%)*
Opportunity to improve own health	151^a^ (94%)	158^a^ (100%)	125 (95%)	239 (99%)	339 (99%)	90 (99%)	309 (97%)	122 (98%)	53 (100%)	*1,586 (98%)*
Reputation of institution and staff	154 (96%)	154 (97%)	124 (95%)	238 (99%)	336 (98%)	86 (95%)	311 (97%)	123 (98%)	52 (98%)	*1,578 (97%)*
Covering medical bills in case of study related injury	144^a^ (90%)	152 (96%)	127 (97%)	237^a^ (98%)	328 (96%)	86 (95%)	303 (95%)	122 (98%)	52 (98%)	*1,551 (96%)*
Receiving results after trial	143^abc^ (89%)	153 (97%)	120^d^ (92%)	237^a^ (98%)	331^b^ (97%)	87 (96%)	303 (95%)	125^cd^ (100%)	52 (98%)	*1,551 (96%)*
Side effect of new treatment	133^abcde^ (83%)	150^a^ (95%)	120 (92%)	228^b^ (95%)	328^c^ (96%)	87 (96%)	303^d^ (95%)	120^e^ (96%)	52 (98%)	*1,521 (94%)*
Open label extension	150 (94%)	151 (96%)	115^a^ (88%)	233^a^ (97%)	319 (93%)	85 (93%)	302 (95%)	114 (91%)	49 (92%)	*1,518 (94%)*
Potential negative impact on health	128^abcdefg^ (80%)	143 (91%)	123^a^ (94%)	222^b^ (92%)	325^c^ (95%)	86^d^ (95%)	298^e^ (93%)	121^f^ (97%)	50^g^ (94%)	*1,496 (92%)*
Distance to travel to trial sites	134^ab^ (84%)	141 (89%)	120 (92%)	233^ac^ (97%)	311 (91%)	90^bd^ (99%)	285^cd^ (89%)	113 (90%)	48 (91%)	*1,475 (91%)*
Friendliness of staff	128^ab^ (80%)	145^a^ (92%)	107^c^ (82%)	229^bcd^ (95%)	304 (89%)	76^d^ (84%)	281 (88%)	110 (88%)	48 (91%)	*1,428 (88%)*
Frequency and time spent on clinical visits	132 (83%)	143 (91%)	111 (85%)	216 (90%)	302 (88%)	85 (93%)	286 (90%)	102 (82%)	48 (91%)	*1,425 (88%)*
Physician's recommendations	126 (79%)	141 (89%)	119 (91%)	192 (80%)	294 (86%)	72 (79%)	277 (87%)	113 (90%)	44 (83%)	*1,378 (85%)*
Privacy and confidentiality	91^abcdefgh^ (57%)	137^a^ (87%)	114^b^ (87%)	215^ci^ (89%)	277^d^ (81%)	75^e^ (82%)	247^fi^ (77%)	104^g^ (83%)	49^h^ (92%)	*1,309 (81%)*
Keeping current doctor during trial	103^abcde^ (64%)	126 (80%)	110^a^ (84%)	191^b^ (79%)	281^c^ (82%)	65 (71%)	248^d^ (78%)	109^e^ (87%)	43 (81%)	*1,276 (79%)*
Possibility of getting placebo	95 (59%)	103 (65%)	87 (66%)	157 (65%)	235 (69%)	52 (57%)	188 (59%)	91 (73%)	38 (72%)	*1,046 (64%)*
Payment to participate	30^abcdefg^ (19%)	95^a^ (60%)	83^bhi^ (63%)	128^c^ (53%)	148^dhj^ (43%)	47^ek^ (52%)	100^ijklm^ (32%)	72^fl^ (58%)	31^gm^ (58%)	*734 (45%)*

Note: % endorsing “Somewhat” and “Very important”. Significant pairwise difference between classes were tested by the Fisher’s test with bootstrap adjusted *p*-values for pairwise comparisons (*p* < .05), denoted by the same alphabetic superscript in each row

### Condition-specific attitudes toward trials

The perceptions of ALS patients were significantly different from other patients on a number of factors, suggesting that patients with this more serious and lethal disease would be more accommodating around issues of side effects, privacy and confidentiality, having their bills covered, receiving results after the study, changing doctors during the trial, and not receiving remuneration for taking part. For nearly every other factor, all non-ALS conditions were in agreement. However, there were more between-group differences on the issue of being paid to participate in a trial, with higher levels of interest expressed by patients with epilepsy (63%), type II diabetes (60%), RA (58%), and SLE (58%) relative to patients with Parkinson’s (32%) or ALS (19%). Three of the four groups expressing an interest in being paid were also those with the lowest NPS score.

## Discussion

In a survey of over 1,600 patients with chronic illnesses, we found a high degree of willingness to take part in trials but a low degree of trial experience and variable rate of trial satisfaction among those that had ever taken part. Online platforms might provide a means for identifying and perhaps even resolving some of the challenges to recruitment and retention before they happen by asking patients to partner with researchers to improve their studies [16]. in a study of cancer trials in the UK, studies that included patient input were twice as likely to successfully hit their recruitment targets [20]. Use of the NPS might provide an opportunity to benchmark trials and see whether patient input improves satisfaction. Although not directly comparable, a recent cross-industry survey suggests that currently, the level of patient satisfaction ascribed to trials is only comparable in the consumer world to some of the worst experiences reported in the field [21]; dealing with internet service providers, utility companies, or health insurers are often held up as unpleasant ordeals and this data speaks to substantial room for improvement if we are to encourage participants to enroll.

The main barrier to taking part in trials appeared to be a lack of awareness, with most patients (61%) not being invited to take part by their physicians. Clinic appointments for complex chronic conditions are often too brief to adequately manage all aspects of care, coordination, education, and documentation, let alone to also perform adequate informed consent procedures for a detailed protocol. Infrastructure issues may be important here; McDonald et al. (2006) found that the most important predictor of successful trial recruitment was having a dedicated trial manager (OR 3.8, 95%CI 0.79–36.14). Embi et al. [22] found significant improvements when an automated software system was integrated with electronic health records, and the increasing move towards electronic systems for records management might be one opportunity to match eligible participants to enrolling trials. Disease advocacy groups may also facilitate enrollment with patient-facing features such as clinical trial awareness meetings and advocacy initiatives [23] or online tools such as the Michael J Fox Foundation’s Fox Trial Finder [24]. PatientsLikeMe provides its members an automated tool that matches them to trials for which they might be eligible on the basis of public data from ClinicalTrials.gov, although a recent review found that this data source itself might be less than perfect for such purposes [25].

In line with the literature [26, 27] females and non-whites were under-represented amongst the patients who had experience of being in a trial. Patients in hard-to-reach group may need further outreach, such as meeting patients where they are at major community events, churches, senior centers, and schools (Institute of Medicine, 2012). Once participation was offered, commonly cited reasons for declining to participate were factors such as inconvenience to travel sites, concerns over side effects, and chance of getting a placebo. Given that these research-related factors might be a deterrent to participation, researchers could consider more patient-centered trial designs that emphasize at-home testing, put systems in place to help patients report and cope with side effects, or use novel designs such as adaptive or pragmatic trials to reduce use of placebo. Communicating these patient-centric innovations concisely in recruitment materials could be a key part of stimulating interest in a new study; sometimes the features of a clinical trial are so obscured in recruitment materials that patients incorrectly infer that benefits such as out-of-pocket expenses are not covered when they are [28].

Previous studies have found that altruism and personal benefits are among the key motivators for participating in trials generally [29] and our study replicated this finding. From the patient standpoint, the majority of participants cited that it was also important to get medical bills covered (96%) or get the trial results once the trial was over (95%), and there is some evidence to suggest that in practice most trial participants never receive this feedback [30]. Understanding and addressing such patient needs and expectations are critical steps forward towards designing patient-centered trials rather than assuming that altruism alone will drive recruitment efforts.

In the current study, ALS patients were less concerned about undesirable aspects of trials such as potential negative impact on health and side effects of new treatment. One possible explanation of these findings is that due to the lack of disease-modifying therapeutics and reduced life expectancy, ALS patients may be more desperate to find a cure. Other studies considering chronic conditions have found that cancer trials were more likely to fulfill their recruitment targets, for instance [20]. Previous research has found that ALS patients might be particularly unusual in the lengths that they will go to self-experiment, including off-label treatments and even breaking trial protocols to attempt to deduce whether an experimental treatment is having an effect [16].

Patients with the rarer conditions ALS, PD or MS were more experienced with trials (36%, 36 and 20% respectively) than patients with the more common T2DM, fibromyalgia and MDD (13%, 11 and 11%). This may be a reflection of different factors such as (1) the perceived severity of their condition; (2) availability of available therapies, (3) opportunity to choose between trials in the investigational pipeline, (4) inherent disease characteristics such as exclusionary comorbidities, and (5) engagement with organizations supporting research. Given that PatientsLikeMe has historically had deep and engaged communities in ALS, PD, and MS, this may simply reflect unusually high levels of engagement in these respondent. However, it might be worth briefly considering the conditions with lower levels of trial participation. T2DM patients might be less willing to participate in clinical research because the perceived threat from the illness is lower than some of the other conditions profiled here. Fibromyalgia trials suffer from an FDA-mandated discontinuation of all other therapies, with the exception of acetaminophen, as a requirement for trial participation, which 84% of patients rated as undesirable [31]. Mental health disorders such as MDD have previously been identified as challenging to enroll in clinical trials due to stigma, trial issues arising from patients with a heightened risk of suicide, and the availability of existing therapies [32]. Once enrolled, however, MDD patients in this study reported some of the highest levels of satisfaction with their trials.

The merits of the study include utilizing crowdsourced PPRNs for rapid enrollment and data collection in a group of patients who have either been in clinical trials or who have a high degree of interest in taking part in them. These networks hold promise for optimizing trial design and operations by enabling researchers to evaluate to patients’ concerns in a way that is fast, repeatable, and with results that can generalize to a broader population easier than individual advocates or small focus groups. Furthermore, given the participatory nature of our research, this provides a framework to re-engage patients for future feedback and evaluate the disease correlates (e.g., severity, treatment use) that may predict trial preferences in the future. This framework can be adapted for future studies to elicit trial-specific patient-centered feedback in real-time that can not only be targeted to improve recruitment but also ensure that patient expectations are met from trials.

There are several limitations to the current study design. The study was cross-sectional survey and hence subject to selection bias, recall bias, information bias and social desirability bias. It may represent the patient voice, but the data are based on subjective self-reports, the data are derived from a convenience sample, and the mix of patients is representative of PLM and not necessarily of the medical population at large. The PLM population skews toward a more activated, educated, female population with chronic conditions, although this is typical for many health-oriented sites [33, 34]. Finally, condition comparisons presented in the study were not specified a-priori. While adjustments were made for multiple pairwise comparisons, statistics are purely exploratory.

## Conclusions

Patients are willing and able to take part in clinical trials for altruistic reasons but are rarely given the opportunity to do so. Once enrolled, they face an experience that could be improved upon by consulting with other patients like them to improve protocol design. Although patient factors are only one aspect of conducting a successful trial there are sound ethical, scientific, and business reasons for taking a more patient-centric approach.

## Additional file

Additional file 1: Appendix: Survey Questionnaire (DOCX 16 kb)

## Abbreviations

ALS: Amyotrophic lateral sclerosis
CI: Confidence interval
FDA: Food and Drug Administration
FM: Fibromyalgia
MDD: Major depressive disorder
MS: Multiple sclerosis
NPS: Net Promoter Score
OHRP: Office for Human Research Protections
OR: Odds ratio
PD: Parkinson disease
PLM: PatientsLikeMe
PPRN: Patient powered research network
RA: Rheumatoid arthritis
SLE: Systemic lupus erythematous
T2DM: Type 2 diabetes

## Glossary

Primary condition: Is what patients are most interested in learning about at PatientsLikeMe; members have the option to add multiple conditions and comorbidities to their profiles in addition to primary condition.
Net Promoter Score (NPS): A single item measure of consumer satisfaction used to measure performance (0–6: detractors, 7–8: passive and 9–10: promoters). NPS has been used as an overarching measure of patient experience with healthcare delivery [18]. Patients were asked “How likely would you be to recommend taking part in this specific trial to another eligible patients like you?”.
Trial experience: Defined as patients who partook in a trial (regardless of completion) or were participating at the time of the survey.

## References

[1] Collier R (2009). Rapidly rising clinical trial costs worry researchers. CMAJ.

[2] DiMasi JA, Grabowski HG, Hansen RW (2015). The cost of drug development. N Engl J Med.

[3] Dickson S, Logan J, Hagen S (2013). Reflecting on the methodological challenges of recruiting to a United Kingdom-wide, multi-centre, randomised controlled trial in gynaecology outpatient settings. Trials.

[4] Robiner WN, Yozwiak JA, Bearman DL, Strand TD, Strasburg KR (2009). Barriers to clinical research participation in a diabetes randomized clinical trial. Soc Sci Med.

[5] Allison M (2009). Can web 2.0 reboot clinical trials?. Nat Biotechnol.

[6] Stevens Z, Carpenter H, Gawler S (2013). Lessons learnt during a complex, multicentre cluster randomised controlled trial: the ProAct65+ trial. Trials.

[7] Discovery F on D, Development, Translation and, Policy B on HS, Medicine I of. Public Engagement and Clinical Trials: New Models and Disruptive Technologies: Workshop Summary. National Academies Press; 2012. https://books.google.com/books?id=00yfAwAAQBAJ&pgis=1. Accessed 30 Sept 2015. 22514814

[8] Brintnall-Karabelas J, Sung S, Cadman ME, Squires C, Whorton K, Pao M (2011). Improving recruitment in clinical trials: Why eligible participants decline. J Empir Res Hum Res Ethics An Int J.

[9] Frank G. Current Challenges in Clinical Trial Patient Recruitment and Enrollment. 2004

[10] Getz KA, Wenger J, Campo RA, Seguine ES, Kaitin KI (2008). Assessing the impact of protocol design changes on clinical trial performance. Am J Ther.

[11] Mullins CD, Abdulhalim AM, Lavallee DC (2012). Continuous patient engagement in comparative effectiveness research. JAMA.

[12] De Wit M, Abma T, Koelewijn-van Loon M, Collins S, Kirwan J (2013). Involving patient research partners has a significant impact on outcomes research: a responsive evaluation of the international OMERACT conferences. BMJ Open.

[13] Perlmutter J (2011). Advocate Involvement in I-SPY 2. Breast Dis A Year B Q.

[14] A Tufts Center for the Study of Drug Development. Industry Usage of Social and Digital Media Communities in Clinical Research. 2014. http://csdd.tufts.edu/files/uploads/TCSDD_Social_Media_Final.pdf. Accessed 2 Nov 2015

[15] Getz KA, Zuckerman R, Cropp AB, Hindle AL, Krauss R, Kaitin KI (2011). Measuring the incidence, causes, and repercussions of protocol amendments. Ther Innov Regul Sci.

[16] Wicks P (2014). Could digital patient communities be the launch pad for patient-centric trial design?. Trials.

[17] Brownstein CA, Brownstein JS, Williams DS, Wicks P, Heywood JA (2009). The power of social networking in medicine. Nat Biotechnol.

[18] Hamilton DF, Lane JV, Gaston P (2014). Assessing treatment outcomes using a single question: the Net Promoter Score. Bone Joint J.

[19] Galesic M (2006). Dropouts on the Web: effects of interest and burden experienced during an online survey. J Off Stat.

[20] McDonald AM, Knight RC, Campbell MK (2006). What influences recruitment to randomised controlled trials? A review of trials funded by two UK funding agencies. Trials.

[21] Net Promoter Score Benchmark Study, 2014. http://www.temkingroup.com/research-reports/net-promoter-score-benchmark-study-2014/. Accessed 30 Sept 2015.

[22] Embi PJ, Jain A, Clark J, Bizjack S, Hornung R, Harris CM (2005). Effect of a clinical trial alert system on physician participation in trial recruitment. Arch Intern Med.

[23] Mathur S, DeWitte S, Robledo I, Isaacs T, Stamford J (2015). Rising to the challenges of clinical trial improvement in Parkinson’s disease. J Parkinsons Dis.

[24] Rocker C, Cappelletti L, Marshall C (2015). Use of an online portal to facilitate clinical trial recruitment: a preliminary analysis of Fox Trial Finder. J Parkinsons Dis.

[25] Pfiffner PB, Oh J, Miller TA, Mandl KD (2014). ClinicalTrials.gov as a data source for semi-automated point-of-care trial eligibility screening. Raghava GPS, ed. PLoS One.

[26] Lovato LC, Hill K, Hertert S, Hunninghake DB, Probstfield JL (1997). Recruitment for controlled clinical trials: literature summary and annotated bibliography. Control Clin Trials.

[27] Baquet CR, Commiskey P, Daniel Mullins C, Mishra SI (2006). Recruitment and participation in clinical trials: socio-demographic, rural/urban, and health care access predictors. Cancer Detect Prev.

[28] Bedlack RS, Wicks P, Heywood J, Kasarskis E (2010). Modifiable barriers to enrollment in American ALS research studies. Amyotroph Lateral Scler.

[29] Ellis PM, Butow PN, Tattersall MH, Dunn SM, Houssami N (2001). Randomized clinical trials in oncology: understanding and attitudes predict willingness to participate. J Clin Oncol.

[30] Cox K, Moghaddam N, Bird L, Elkan R (2011). Feedback of trial results to participants: a survey of clinicians’ and patients’ attitudes and experiences. Eur J Oncol Nurs.

[31] Holman AJ, Neradilek MB, Dryland DD, Neiman RA, Brown PB, Ettlinger RE (2010). Patient-derived determinants for participation in placebo-controlled clinical trials for fibromyalgia. Curr Pain Headache Rep.

[32] (US) I of M. Public Engagement and Clinical Trials. 2012. http://www.ncbi.nlm.nih.gov/books/NBK91498/. Accessed 30 Sept 2015.

[33] Thackeray R, Crookston BT, West JH (2013). Correlates of health-related social media use among adults. J Med Internet Res.

[34] Kontos E, Blake KD, Chou W-YS, Prestin A (2014). Predictors of eHealth usage: insights on the digital divide from the Health Information National Trends Survey 2012. J Med Internet Res.

