# Supplementary material for: Clinical trials from the patient perspective: survey in an online patient community
Source: BMC Health Serv Res. 2017 Feb 27;17:166. doi: 10.1186/s12913-017-2090-x (PMC5327530; doi:10.1186/s12913-017-2090-x)
Supplement: Additional file 1: — Appendix: Survey Questionnaire (DOCX 16 kb) [file 12913_2017_2090_MOESM1_ESM.docx]

## Appendix: Survey Questionnaire

1. **Basic Info (standard questions with profile import) [all]**
   1. DOB
   2. Sex
   3. Location
   4. ZIP Code
   5. Race
   6. Ethnicity
2. **Trial Awareness [all]**
   1. TRIALDISCUSS - Has your doctor or other health care professional ever talked to you about medical research? [Yes, No, Not sure]
   2. TRIALDOCTORREC - If your doctor found a clinical trial for you and recommended you join; how likely would you be to participate in a clinical trial? [Very likely, somewhat likely, not likely, would not participate, not sure]
   3. TRIALSCREEN - Have you ever been asked to participate in a clinical trial? [Yes/No]
3. **Trial Participation** [If TRIALSCREEN = yes]
   1. TRIALDECISION - When you were asked to participate in the trial, what happened? *If you have been asked to participate in more than one clinical trial, please think about just the most recent one.*
      1. I took part and am still in the trial
      2. I took part and completed the trial
      3. I took part but withdrew before the end
      4. I wanted to take part but was not eligible
      5. I declined to take part in the trial

- 1. TRIALINFO - How did you find out about this clinical trial? *If you have been asked to participate in more than one clinical trial, please think about just the most recent one.*
     1. PatientsLikeMe
     2. Online/internet
     3. Media
     4. Advertisements
     5. Doctor / healthcare provider
     6. Friends/Family
     7. Patient Organization
     8. Other
     9. Not sure

1. **Trial Accepted Details** [If TRIALDECISION = i-iii]

*You told us in an earlier question that you started a tria*l. If you have taken part in more than one, please tell us about the most recent trial that you started in the questions that follow.

- 1. TRIALCONSENTDATE - Roughly when did you agree to take part in the trial? *Your best guess is fine* [Fuzzy date]
  2. TRIALSTARTDATE - Roughly when did you first get treatment in the trial? *Your best guess is fine* [Fuzzy date]
  3. DROPOUTCONSIDERED - Thinking about this most recent trial, did you ever *consider* withdrawing your consent, dropping out, or leaving the clinical trial early?
     1. Yes
     2. No
  4. In your lifetime, in how many trials have you taken part? *Please enter numbers only, your best guess is fine* [number]

1. **Trial Considered Dropout Open Text** [if DROPOUTCONSIDERED = Yes]

*You told us in an earlier question that you considered withdrawing your consent, dropping out, or leaving the trial early*

- 1. DROPOUTCONSIDEREDFREETEXT - Why did you consider leaving the clinical trial?  *Please explain in as much detail as possible* [open text]

1. **Trial Accepted Recal**l [If TRIALDECISION = i-iii]

*You told us in an earlier question that you started in a clinical trial*. In each of the following questions we will ask you to remember several aspects of the trial - your best guess is absolutely fine, or if you don’t remember please just write “can’t remember”

- 1. What was the name of the experimental treatment or the trial in which you took part? [Free text]
  2. What were the best things about taking part in this trial? [Free text]
  3. What were the worst things about taking part in this trial? [Free text]
  4. What were you told about the results of the trial? [Free text]
  5. Overall, how satisfied were you with the trial? (1 – Not at all satisfied, 2 – slightly satisfied, 3 – moderately satisfied, 4 – Very satisfied, 5 – Extremely satisfied)
  6. How likely would you be to recommend taking part in this specific trial to another eligible patient like you? [Net promoter score 0-10]
  7. How likely would you be to encourage taking part in clinical trials in general to another patient? [Net promoter score 0-10]

1. **Trial Declined Open Text** [If TRIALDECISION = v. I declined to take part in the trial]

*You told us in an earlier question that you declined to take part in the trial*

- 1. DECLINEFREETEXT Why did you decide not to participate in the clinical trial? Please explain in as much detail as possible [open text]

1. **Trial Declined Fixed Choices** [If TRIALDECISION = v. I declined to take part in the trial]

*Thank you for sharing your experiences in the previous section. Now we’d also like to ask you to choose from a list of reasons we think people sometimes decline to participate. Your answer on the previous question and this one will help us understand your decision in different ways.*

- 1. DECLINEREASONS - Why did you decline to participate in the trial? *Please check all that apply.*
     1. I am not interested in taking part in any trial at all
     2. I was not interested in this particular trial
     3. I work so I didn’t think I could get the time off work
     4. I was worried about the side effects of the trial
     5. I did not want to take part if there was a chance I might be given placebo
     6. I did not trust the motivations of the study sponsor / product manufacturer
     7. It did not offer enough financial compensation for my time and expenses
     8. I felt too unwell to take part
     9. My friends or family advised me not to
     10. It was inconvenient for me to travel to the trial sites

1. **Trial Non-Completer Detail**s [If TRIALDECISION = v]

*You told us in an earlier question that you withdrew your consent, dropped out, or left the trial early*

- 1. DROPOUTFREETEXT - Why did you leave the clinical trial early?  *Please explain in as much detail as possible* [open text]

1. **Interest in Trials [all, Question_Matrix]**

We are interested in your attitudes towards taking part in trials. Please consider each statement below and rate how strongly you agree or disagree.

- 1. INTERESTTRIAL - I am interested in learning more about taking part in trials [Strongly agree, agree, disagree, strongly disagree]
  2. INTERESTTRIAL12MTHS - I would like to take part in a clinical trial in the next 12 months [Strongly agree, agree, disagree, strongly disagree]
  3. INTERESTPLMTRIALS - If PatientsLikeMe offered a feature on the website that allowed me to easily connect to trials that are a good match, I would use it [Strongly agree, agree, disagree, strongly disagree]
  4. INTERESTHELPDESIGN - In the future, I would be interested in helping researchers to design better trials by answering questions about the design of their trial [Strongly agree, agree, disagree, strongly disagree]

1. **Trial Factors [all]**

If you were thinking about joining a new clinical trial, how important would the following factors be in your consideration? [Very important, somewhat important, not very important, not at all important, not sure]

- 1. The potential negative impact the trial could have on my health
  2. The distance I would have to travel for my trial visits
  3. Keeping my current doctor during the trial
  4. The side effects that might come from being on a new treatment
  5. The possibility that I might be given a placebo (inactive treatment)
  6. The friendliness of the clinical and researchers
  7. The number of visits and total time per month to participate
  8. Being given the results of my trial after my participation had ended
  9. Having the option to continue on the new treatment after the trial had concluded
  10. The reputation of people or the institution conducting the research
  11. Whether I would have medical bills covered if I had an injury from the study
  12. An opportunity to possibly improve my own health
  13. Privacy and confidentiality issues
  14. The opportunity to improve the health of others
  15. My physician’s recommendation
  16. Whether I would be paid to participate

1. **Finale**

*Thank you for taking the time to participate in this study - we will be analyzing your responses and hope to have some responses to share with you over email in the next 6-8 weeks*

- 1. FINALE - If you have any other comments about this survey or your experience with trials in general please enter them in the box below otherwise hit “Submit Survey” to complete the study. Thanks again! [Free text]
